# Assembly of the Novel Five-Component Apicomplexan Multi-Aminoacyl-tRNA Synthetase Complex Is Driven by the Hybrid Scaffold Protein Tg-p43

**DOI:** 10.1371/journal.pone.0089487

**Published:** 2014-02-20

**Authors:** Jason M. van Rooyen, Jean-Benjamin Murat, Pierre-Mehdi Hammoudi, Sylvie Kieffer-Jaquinod, Yohann Coute, Amit Sharma, Hervé Pelloux, Hassan Belrhali, Mohamed-Ali Hakimi

**Affiliations:** 1 European Molecular Biology Laboratory, Grenoble, France; 2 CNRS, UMR5163, LAPM, Grenoble, France; 3 Université Joseph Fourier, Grenoble, France; 4 CEA, IRTSV, Laboratoire Biologie à Grande Echelle, Grenoble, France; 5 Structural and Computational Biology Group, International Centre for Genetic Engineering and Biotechnology (ICGEB), New Delhi, India; University at Buffalo, United States of America

## Abstract

In *Toxoplasma gondii*, as in other eukaryotes, a subset of the amino-acyl-tRNA synthetases are arranged into an abundant cytoplasmic multi-aminoacyl-tRNA synthetase (MARS) complex. Through a series of genetic pull-down assays, we have identified the enzymes of this complex as: methionyl-, glutaminyl-, glutamyl-, and tyrosyl-tRNA synthetases, and we show that the N-terminal GST-like domain of a partially disordered hybrid scaffold protein, Tg-p43, is sufficient for assembly of the intact complex. Our gel filtration studies revealed significant heterogeneity in the size and composition of isolated MARS complexes. By targeting the tyrosyl-tRNA synthetases subunit, which was found exclusively in the complete 1 MDa complex, we were able to directly visualize MARS particles in the electron microscope. Image analyses of the negative stain data revealed the observed heterogeneity and instability of these complexes to be driven by the intrinsic flexibility of the domain arrangements within the MARS complex. These studies provide unique insights into the assembly of these ubiquitous but poorly understood eukaryotic complexes.

## Introduction

Aminoacyl-tRNA synthetases (aaRSs) are responsible for correctly charging tRNA molecules with their cognate amino acids, the essential precursor step of protein translation in all life [1]. While certain aaRSs are absent in prokaryotes, eukaryotes on the other hand possess a full complement of the 20 synthetases and these often contain additional non-catalytic regions, making them on average larger than their prokaryotic counterparts [2]. It is these N- and C-terminal extensions, and their derivatives, that endow the eukaryotic aaRSs with their non-canonical functions including their roles as cytokines responsible for eliciting inflammatory and apoptotic responses [3], [4].

In eukaryotes a subset of these aaRSs are arranged into a stable cytoplasmic assembly called the multi-aminoacyl-tRNA synthetase complex (MARS). The largest and best described complex is found in higher eukaryotes, from vertebrates to arthropods, and comprises nine aaRSs from both class I and II: aspartyl (DRS), lysyl (KRS), glutaminyl (QRS), methionyl (MRS), arginyl (RRS), leucyl (LRS), isoleucyl (IRS), and the bifunctional glutamyl-prolyl aminoacyl-tRNA synthetase (ERS,PRS), together with three accessory proteins: p18, p38, and p43 [5]. As in the case of the aaRSs, some of these aminoacyl-tRNA synthetase-interacting multifunctional proteins (AIMPs) have regulatory roles as cytokines or precursors thereof [6]. Notably, p43 is known to be cleaved into EMAP II, a secreted peptide implicated in apoptosis induction and angiogenesis [7]. Detailed protein-protein interaction studies [8] however have shown that the most important roles of the AIMPs is as the principal factors driving the assembly of MARS complexes. Specifically, it is now understood that dimers of the p18 and p43 scaffold proteins are each responsible for the assembly of separate subcomplexes which are then brought into association by their interactions with a dimer of p38 [9]. In addition to the interactions affected by the scaffold proteins there is also strong evidence for direct associations between aaRSs [10]. Surprisingly, the MARS complex from the nematode *Caenorhabditis elegans* differs by the absence of the p18 and p43 scaffold proteins, and only contains eight of the nine aaRS (seven of which are common) present in the human complex [11]. In addition to the assemblies discovered in metazoans, a rather limited and well characterized MARS complex has been isolated from the yeast *Saccharomyces cerevisiae*. It is composed of only the class I aaRSs: methionyl- and glutamyl-RS, together with one scaffold protein, Arc1p [12].

Although several theories have been suggested for the function of these assemblies, the best supported being (a) direct channeling of charged tRNA to actively translating ribosomes via nuclear export of charged tRNA [13], [14], (b) the enhancement of aaRS activity through the recruitment of non-specific tRNA binding domains [15], and (c) spatio-temporal control of the moonlighting functions of component aaRSs [9], the exact cellular role and the basis for the choice of aaRS in the complex remains in dispute. In spite of this uncertainty and the differences in both the arrangements and numbers of components present in the various MARS complexes described to date, common features do suggest a shared mechanism of assembly. In particular, several conserved domains have been identified in both the non-catalytic extensions of the aaRSs and the AIMPs [4]. For instance, a motif with homology to the C-terminal domain of glutathione-S-tranferase (GST-like domain) occurs frequently in proteins associated with translation [5] and is known to drive pairwise protein-protein interactions in these assemblies [16]. Another commonly observed motif derives from ancestral tRNA binding proteins, like Trbp111, and in addition to their role in enhancing the catalytic activity of binding partners, through non-specific tRNA binding [17], [18], these motifs have also been implicated in protein associations [19]. The molecular basis underlying these interactions has best been described in the yeast MRS-ERS-Arc1p MARS complex. Through a series of structural and biochemical studies it has been shown that the GST-like domain of the hybrid Arc1p scaffold protein is solely responsible for the associations with the aaRSs of the complex [20] and that these dimerization interfaces are contributed by independent sites in the same motif [21]. Whether similar interfaces are responsible for some interactions of components in the larger metazoan complexes is highly likely but the exact molecular basis of all the interactions and how they relate to the function of these poorly understood complexes still awaits further structural studies.

In an attempt to answer these questions and to shed light on the universality of the current assumptions regarding these apparently ubiquitous eukaryotic complexes, we undertook a molecular interaction, biochemical, and ultra-structural study of a MARS complex from *Toxoplasma gondii.* In this work, we provide the first description of the composition and arrangement of the MARS complex from apicomplexans, a phylum of unicellular eukaryotes that comprises several obligate intracellular parasites of medical importance, and also explain some of its properties, such as its instability and heterogeneity, directly in terms of the flexibility of its structure. Our results have important implications for future structural studies aiming to elucidate the molecular basis of the assembly and functioning of the MARS complex in higher eukaryotes.

## Materials and Methods

### Chemicals and Cell culture methods

All chemicals were sourced through Sigma-Aldrich and cell culture media from Life technologies unless otherwise specified. Human foreskin fibroblast (HFF) and Human Embryonic Kidney 293 cells (HEK293) cell lines were cultured in Dulbecco's modified Eagle's medium supplemented with 10% (v/v) heat-inactivated fetal bovine serum, 10 mM HEPES buffer (pH 7.2), 4 mM glutamine, and 50 mg/mL each of penicillin and streptomycin. Cells were incubated at 37°C with 5% CO_2_ in humidified air. All *Toxoplasma* strains were maintained by serial passage in HFF monolayers.

### Animal methods

Outbred female Swiss mice, weighing approximately 20 g, each were used for *in vivo* experiments. These animals were maintained in specific pathogen-free conditions in accordance with institutional and national regulations and all protocols were approved by the Floralis (Joseph-Fourier University) Committee on Animal Care. Parasites were inoculated by intraperitoneal injection of 200 µL of a suspension in Hank's balanced salt solution (HBSS). To check that an infection had occurred, mice that did not spontaneously die were euthanized in an approved CO_2_ chamber; whole blood was collected through an intracardiac puncture, and serum was subjected to the Toxoscreen DA (bioMérieux) assay. To test the ability to induce cysts, brains from these mice were treated as previously described [22] and cysts were observed and numbered microscopically.

### Bioinformatics

Homologs of the *Toxoplasma* p43 protein (*TgME49_223140*) were identified by GenTHREADER [23] after splitting the sequence into domains on the basis of the NCBI Conserved Domain Search annotation [24]. These included: S*accharomyces cerevisiae* Arc1p (pdb id: 2htqA), *Homo sapiens* p18 (2uz8A), *Homo sapiens* p43 (1fl0A). In order to generate a multiple-sequence alignment, the corresponding structures were aligned in UCSF Chimera [25] using Match-Maker [26]. The Tg-p43 and Hs-p38 sequences were then manually aligned to the resulting structure-based profile, from Match-Align, on the basis of the original GenTHREADER alignment using GeneDoc [27]. The latter program was also used for sequence identity calculations and alignment rendering. Domain structure analyses of the GST C-terminal domain containing proteins: Tg-MRS (*TgVEG_083160*), Tg-QRS (*TgVEG_007070*), Tg-ERS (*TgVEG_049140*), Tg-p43 (*TgME49_223140*), and Tg-YRS (*TGVEG_000460*) were carried out using NCBI Conserved Domain Search with an E-value threshold of 1. *In silico* predictive disorder analysis was carried out with Genesilico MetaDisorder2 [28].

### Construction of recombinantly tagged MARS subunits and Tg-p43 knockout

To construct the vectors: pLIC-p43-Myc-FLAG, pLIC-p43ΔCterm-HA-FLAG, pLIC-p43-HA-FLAG, pLIC-YRS-HA-FLAG, and pLIC-MRS-HA-FLAG, coding sequences of Tg-p43, Tg-YRS and Tg-MRS were amplified from genomic DNA of a RH *ΔKu80* strain using primers (Table S1). The resulting PCR products were cloned into pLIC-MF-dhfr and pLIC-HF-dhfr vectors using the LIC cloning method as described previously [29]. Plasmids were transfected into freshly isolated tachyzoites by electroporation as described previously [29] and transfected parasites were selected for resistance to pyrimethamine (DHFR selection) and cloned by limiting dilution.

To generate a Tg-p43 knockout, the Multisite Gateway Pro 3-fragment Recombination system (Life Technologies) was used to clone the dhfr cassette or the hxgprt cassette flanked by the 5′ and 3′ surrounding regions of the Tg-p43 coding sequence of RH-Δ*Ku80* and Pru Δ*Ku80* genomic DNA as described previously (20) using primers attB1-066670, attB4-066670, attB3-066670 and attB2-066670 (Table S1). The final plasmids (pΔTg-p43-DHFR or pΔTg-p43-HXGPRT) were used as templates to amplify the sequences for transfection, using primers attB1–066670 and attB2–066670. Purified DNA fragments were transfected as above and clones selected for by incorporation of both xanthine and mycophenolic acid (HXGPRT selection).

### Immunoﬂuorescence assays (IFA)

For labeling, HFFs grown on cover-slips infected with parasites were treated as described previously [29] with the following modifications. Coverslips were incubated for 1 h with the primary antibodies anti-Myc (9E10:sc-40, Santa Cruz Biotechnology), anti-HA (3F10; Roche) or anti-TgQRS rabbit polyclonal antibodies (see Western blot section below), followed by the secondary antibodies goat anti-mouse IgG or goat anti-rabbit IgG coupled with either Alexa Fluor 568 or Alexa Fluor 488 dye (Invitrogen) at a 1∶1,000 dilution. *Toxoplasma* anti-TgSUMO labels parasite nucleus [30].

### Immunoprecipitation of recombinantly tagged MARS subunits

Cell-free extracts containing FLAG-tagged proteins were prepared as follows: extracellular parasites from twenty 500 cm^2^ flasks (Nunc TripleFlasks, Thermo Fisher Scientific) were lysed in 7 mL of BC100 buffer (20 mM TrisHCl pH 8, 10% glycerol; 0.2 mM EDTA, 0.1 M KCl) with the addition of a protease inhibitor cocktail (Complete Protease Inhibitor Tablets, Roche) using a Dounce homogenizer (tight plunger). The lysate was centrifuged twice at 21,000 g for 10 min at 4°C. The supernatant was then incubated with 200 µL anti-FLAG M2 affinity gel (Sigma- Aldrich) for 1 hour. Beads were then washed with 5 column volumes (CV) washing buffer (25 mM Tris pH 7.5, 150 mM KCl, 10% glycerol, 10 mM MgCl2), followed by 5 CV of washing buffer with 500 mM KCl and finally another 5 CV of low-salt wash buffer, before bound polypeptides were eluted with two CV of wash buffer containing 250 µg/mL FLAG peptide.

### Size-exclusion chromatography (SEC)

Immunoprecipitation eluates were concentrated to <100 µl by ultra-filtration with a 10 kDA molecular weight cut-off (MWCO) filter (Nanosep centrifugal device, Pall Corporation, Omega membrane) before loading onto a Superdex 200 10/300 GL column (GE Healthcare) using the AKTA Purifier system (GE Healthcare). Separations were carried out in 25 mM Tris pH 7.5, 150 mM KCl, 10% glycerol, 10 mM MgCl_2_ at 4°C with a flow rate of 0.5 mL/min and fractions were collected every minute. The column was periodically calibrated using high-molecular weight standards (Sigma-Aldrich) and the consistency of chromatographic profiles was assessed by comparison of the FLAG peptide's elution volume in each separation.

### Protein analysis, visualization, and identification

Eluates from immunoprecipitation or SEC were separated by sodium dodecyl sulfate-polyacrylamide gel electrophoresis (SDS-PAGE) using pre-cast gradient gels (NuPAGE® Novex®4–12% Bis-Tris polyacrylamide gel, Life Technologies) with MES-SDS running buffer using the XCell SureLock® Mini-Cell as per the manufacturer's instructions. Proteins were then visualized by silver staining using ProteoSilver Plus Silver Stain Kit (Sigma-Aldrich). For identification by mass-spectrometry (MS), proteins were first concentrated by precipitation with trichloracetic acid (1/6th volume), washed twice with ice-cold acetone, before separation by SDS-PAGE and staining with the Colloidal Blue Staining Kit from Life Technologies. Identification of the proteins in the excised bands by MS-MS was carried out as previously described [31].

### Western blot analysis

Cell-free extracts were prepared from three 175 cm^2^ flasks as described above, maintaining the same ratio of resuspension buffer to plate surface area. Following size-exclusion chromatographic separations, eluted fractions were separated by SDS-PAGE and then electro-blotted onto polyvinylidene fluoride membrane (Immobilon-P; Millipore) with the XCell II™ Blot Module (Life Technologies) as per the instructions.

Membranes were then probed with a 1∶4000 dilution of the anti-TgQRS antiserum or a 1∶1000 dilution of an anti-FLAG antibody (mouse Monoclonal ANTI-FLAG® M2 antibody, Sigma-Aldrich). The blots were developed with the SuperSignal West Pico Chemiluminescent Substrate kit (Thermo Fisher Scientific) and imaged on the C-DiGit Blot Scanner (LI-COR Biosciences). The anti-Tg-QRS rabbit polyclonal antibodies were raised against recombinant *E. coli* – derived Tg-QRS protein by Eurogentec following their Speedy 28-day protocol and the final bleed (day 28) was used for blotting.

### Electron Microscopy

Immunoprecipitation eluents of YRS-HA-FLAG, from 17 500 cm^2^ flasks (Nunc TripleFlasks, Thermo Fisher Scientific) using 0.4 µl anti-FLAG resin, were concentrated to 100 µl (from an original volume of 1500 µl) by ultrafiltration with a 10 kDa MWCO centrifugal unit (as described above). Samples were then diluted by 1:5 in 25 mM Tris-HCl pH 7.5, 150 mM NaCl before application of 3.5 µl onto copper electron microscope grids coated with a plasma-treated thin carbon support films (Electron Microscopy Sciences). Staining was accomplished with 2% uranyl acetate solution using the droplet method and electron micrographs were recorded by a Gatan ORIUS 2.7 k×2.7 k CCD camera at a nominal magnification of 25 000 times using a JEOL 1200 EX II microscope operating at 100 kV. Particle images (1030) were manually selected and windowed (128 pixel) from 50 micrographs (sampling of 2.7 Å/pixel) using SIGNATURE [32]. Rotational averages were calculated in EMAN1 [33] following pre-centring and the radial profile was plotted from the resulting total average in ImageJ [34]. Iterative reference-free averaging in EMAN was used to generate the representative average of a manually selected sub-set of images (137 out of 1030) and iterative reference-free classification produced the class averages for heterogeneity assessment.

### Heterologous expression and purification of recombinant proteins

The gene for *Tg-p43* (*TgVEG_053640*), codon-optimized for expression in *E. coli* by GeneArt (Life Technologies), was the starting material for cloning. Primers were designed to amplify a region corresponding to the *TgME49_223140* gene product (see text for details regarding the identification of the shorter gene product and Table S1 for primer sequences) and the included NcoI and BamHI sites were used to subclone the resulting PCR product, which included a C-terminal 6×histidine tag, into a pETDuet-1 (Novagen) expression vector. Positive transformants were selected for in *E. Coli* Top10 cells (Life Technologies) and isolated plasmid DNA was transformed into *E. coli* BL21 (DE3) cells for expression. Cultures were grown in Luria Broth (1 L), at 37°C, with shaking (200 rpm), in the presence of ampicillin, to an optical density of 0.6 (at 600 nm) before Isopropyl β-D-1-thiogalactopyranoside (1 mM final concentration) was added to induce protein expression. Induced cultures were incubated at 37°C for 3 hrs before cells were collected by centrifugation at 5000 rcf for 10 min at 4°C, washed twice in phosphate-buffered saline (PBS), and stored at −20°C. Lysis of cell pellets resuspended in 25 mM Tris pH 7.5, 20 mM imidazole, 300 mM KCl, 10% glycerol, 10 mM MgCl_2_, was accomplished by 3 min sonication (Vibra-Cell VC750 from Sonics and Materials, inc.) on ice before clarification using two rounds of centrifugation at 21000 rcf for 10 min at 4°C. Immobilized metal affinity chromatographic purification was carried out by loading supernatents onto a nickel-nitrilotriacetic acid agarose column (Qiagen) pre-equilibrated with lysis buffer at 1 ml/min. Unbound proteins were removed by washing with 10 column volumes (CV) before elution of tagged-proteins with a linear gradient (10 CV) of imidazole (from 25 mM to 175 mM) in the same buffer. Selected fractions were pooled and concentrated by ultrafiltration (Vivaspin 15 turbo unit – Sartorius-Stedim and Microsep unit – Pall Corporation; 10 kDa MWCO 4°C) to 20 mg/ml before SEC. Following concentration, peak fractions were flash-frozen into liquid nitrogen for long-term storage at –80°C.

The shortened construct of Tg-p43 was also cloned into a pcDNA4 vector (Life Technologies) using primers (Table S1) which included a C-terminal FLAG-tag, as described in [29]. Transient expression was achieved by transfecting HEK293 cells, grown to 60% confluence in 3×75 cm^2^ plates (Greiner Bio-one), with 35 µg of vector DNA and 45 µg of polyethylenimine. Following 48 hrs incubation, with one change of medium at 24 hrs, plates were washed with PBS before storage at −80°C. Cells were then harvested from thawed plates by scraping with 6 ml of 20 mM TrisHCl pH 8, 10% glycerol, 0.2 mM EDTA, 500 mM KCl buffer containing protease inhibitors (Complete Protease Inhibitor Tablets, Roche). Following lysis using a Dounce homogenizer, samples were clarified by two rounds of centrifugation at 21,000 g for 10 min at 4°C. Isolation of tagged protein was achieved with the same protocol as describe above for immunoprecipitation of recombinantly-tagged *Toxoplasma* proteins. For final purification, eluted proteins were concentrated by ultracentrifugation and subjected to SEC.

The sequence for *Tg-QRS* (*TgME49_217460*) was codon-optimized for expression in *E. coli* and synthesized by GenScript. The included NdeI and XhoI restriction sites were used to subclone the gene into a pET-28 b (+) vector (Novagen) and transformants selected in Top10 cells (Life Technologies). Expression and purification conditions were the same as for the Tg-p43 preparations (described above) with the exception that kanamycin was employed during cell culture and cellular lysis was accomplished chemically. Briefly, cell pellets were thawed and resuspended in 1/20^th^ of the volume of 25 mM Tris-HCl pH 8.0, 5 mM EDTA, and 50 mM NaCl containing protease inhibitors (Complete Protease Inhibitor Tablets, Roche). Lysozyme was added to 0.1 mg/ml and samples left shaking at 37°C for 30 min before carrying out three freeze-thaw cycles. Another 30 min incubation followed the addition of MgCl_2_ to 20 mM and DNAse to 0.1 mg/ml.

### Biophysical and biochemical characterization

Analytical SEC separations of recombinantly prepared Tg-p43 from *E. coli* and HEK293 cells were carried out as described above with a calibrated S200 column. Multi-angle laser light scattering measurements were carried out on *E. coli* – derived Tg-p43 samples (2.3 mg/ml), separated by an identical S200 column (operated under the same conditions but without glycerol or DTT and calibrated with the same standards as above) attached to a dedicated chromatography system with an in-line Dawn EOS detector (Wyatt). Circular dichroism spectroscopy analysis was performed on diluted *E. coli* – derived Tg-p43 (2.3 mg/ml in 25 mM phosphate buffer solution containing 150 mM sodium fluoride) at 20°C using a Jasco J810 spectropolarimeter. Cross-linking experiments were carried with purified *E. coli*-derived Tg-p43, β-amylase, and carbonic anhydrase (Sigma HMW SEC standards), following buffer exchange and concentration by ultrafiltration to 1 mg/ml in PBS with 10% glycerol. Cross-linking was carried out with 0.25% glutaraldehyde on ice and the reaction stopped at the given intervals by adding Tris-HCl pH 8 to 300 mM. Ultrafiltration retention tests of *E. coli* -derived Tg-p43 utilized a 100 kDa MWCO filter (Nanosep centrifugal device, Pall Corporation, Omega membrane) operated according to the manufacturer's recommendations.

## Results

### *Toxoplasma* possesses a hybrid p18/p43-like protein, Tg-p43

**Figure 1:**
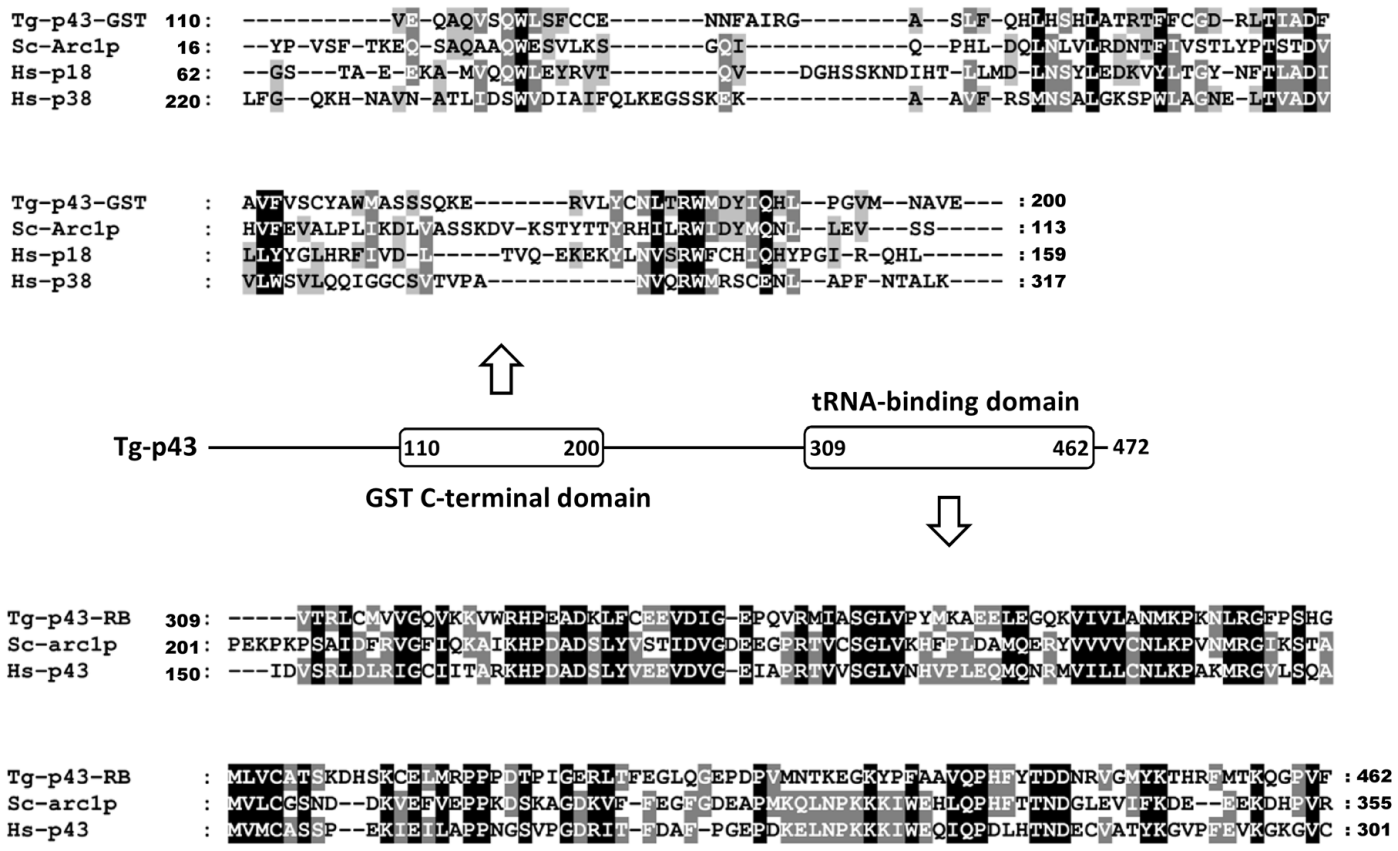
Relationship of Tg-p43 to similar amino-acyl tRNA synthetase-interacting multifunctional proteins from other species. Multiple sequence alignments of the GST-C (GST) and RNA-binding (RB) domains of Tg-p43 and select homologs (Tg = *Toxoplasma gondii*, Sc = *Saccharomyces cerevisiae, Hs = Homo sapiens*) are shown above and below a schematic diagram of the Tg-p43 domain arrangement. The residue numbers of domain boundaries are listed in the schematic and at the beginning and end of the individual sequences.

Our interest in Tg-p43 initially derived from its inclusion in the category of disordered proteins with possible roles in host cell post-translational control. Preliminary isolations of endogenous Tg-p43 (tagged with a FLAG motif via homologous recombination) however soon showed that the EMAPII cytokine-like portion of the protein was not cleaved and secreted beyond the parasitophorous vacuole by tachyzoites, as first suspected. Instead, a highly abundant full-length protein was recovered and confirmed by mass-spectroscopy to correspond to the annotated gene product TgGT1_223140. This protein comprises an all-alpha helical glutathione-S-transferase C-terminal-like domain followed by an EMAPII–like tRNA binding domain (Figure 1). The former domain shares 17% identity with the GST domain of human p18 and only 15% with human p38. The latter domain displays 30% identity with the tRNA-binding domain of human p43 (Figure 1). Tg-p43 also shares 20 and 25% identity with the GST-C and RNA-binding domains respectively of *S. cerevisiae* Arc1p. Being a member of the Myf tRNA-binding domain family, the EMAPII–like tRNA binding domain of Tg-p43 also displays some (13 %) resemblance to the tRNA binding domain of *Toxoplasma* MRS.

**Figure 2:**
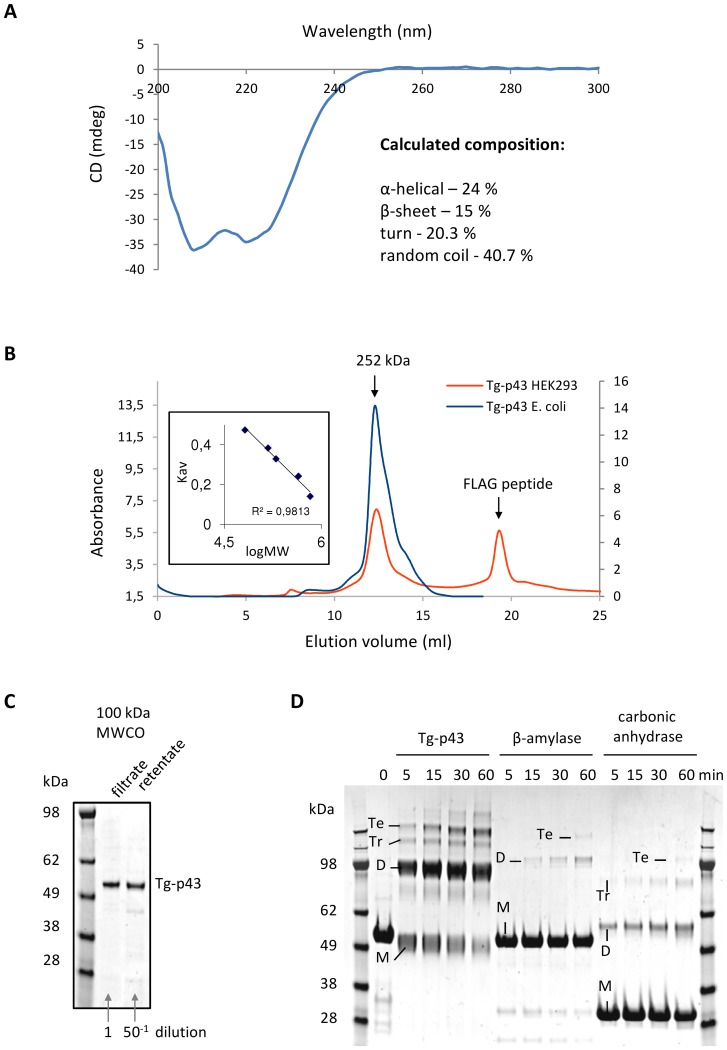
Characterization of the folding and oligomeric state of recombinant Tg-p43. (A) Circular dichroism spectrum of recombinant Tg-p43 expressed and purified from *E. coli*. The percentages of the calculated secondary structures are given in the inset. (B) SEC profile of pure recombinant *E. coli* (blue) and HEK293 (orange)-derived Tg-p43 protein. The MW of the eluted proteins, calculated by calibration with HMW standards (inset left), is shown above the trace. (C) Coomassie blue-stained SDS-PAGE gel image of *E.coli*-derived Tg-p43 retained and passed by a 100 kDa MWCO ultrafiltration device. (D) Coomassie blue-stained SDS-PAGE gel comparing the susceptibility to cross-linking (glutaraldehyde) of *E.coli*-derived Tg-p43 vs. beta-amylase (tetrameric oligomer) and carbonic anhydrase (monomeric) proteins. Based on size considerations, bands have been labeled as either monomers (M), dimers (D), trimers (Tr), or tetramers (Te).

The regions outside of the GST and tRNA-binding domains of the Tg-p43 sequence (38% of the total residues) were predicted to be disordered on the basis of their high percentage of charged and polar residues and this was subsequently confirmed by CD spectroscopy of pure Tg-p43, recombinantly expressed in *E. coli*, which measured a random coil composition of 40.7% (Figure 2A). Interestingly, various biochemical and biophysical analyses of recombinant material purified from both *E. coli* and human (HEK293 cells) expression systems also suggested a tendency of Tg-p43 to oligomerise. Firstly, size-exclusion chromatographic separations of recombinant Tg-p43 from both *E. coli* and human HEK293 cells (Figure 2B), as well as Tg-p43-Myc-FLAG isolated from extracellular parasites (see below) yielded molecular weight estimates far in excess (250 kDa) of the monomeric molecular weight (51 kDa). Secondly, *E. coli*-derived recombinant Tg-p43 is retained by membrane filters with a MWCO of 100 KDa (Figure 2C), and, lastly, Tg-p43 is rapidly chemically cross-linked as a predominantly dimeric species (Figure 2D).

### Tg-p43 is not essential for survival or pathogenesis

In order to test whether p43 is an essential protein in *T. gondii*, the corresponding gene was deleted in the type I strain RHΔKu80 as well as in the type II strain, PruΔKu80 (Figure S1). Both RHΔKu80Δp43 and PruΔKu80Δp43 appeared to be viable without any obvious defect in culture. Most importantly, no differences in infectivity and lethality were observed between RHΔKu80Δp43 or PruΔKu80Δp43 and their parental strains when injected into mice (at lethal inoculums) and their survival monitored (Figure S2). Similarly, numbering of brain cysts after infection with 10^5^ tachyzoites showed that cystogenicity of PruΔKu80Δp43 was maintained (data not shown).

### The *Toxoplasma* MARS complex is cytoplasmic and comprises Tg-p43 together with four aaRSs

**Figure 3:**
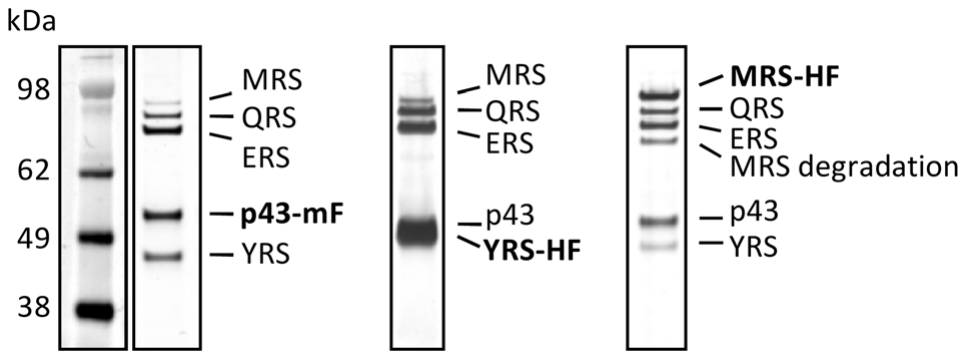
Composition of the *Toxoplasma* MARS complex. Silver-stained PAGE of MARS complex proteins (identified by MS-MS) isolated by FLAG immuno-precipitation of endogenously tagged subunits (indicated in boldface: mF = Myc-FLAG and HF = HA-FLAG epitope tags) from extracellular parasites.

In order to determine if Tg-p43 is involved in *Toxoplasma* MARS complex formation, as predicted by the similarities with the MARS accessory proteins from yeast and humans, binding partners of Myc-FLAG-tagged-Tg-p43 were identified by immunoprecipitation. In addition to the tagged Tg-p43 protein four additional proteins were detected by SDS-PAGE (Figure 3) and identified by MS-MS as: methionyl-tRNA synthetase (106 kDa), glutaminyl-tRNA synthetase (96 kDa), glutamyl-tRNA synthetase (88 kDa), tyrosyl-tRNA synthetase (YRS) (48 kDa). The same result was obtained from freshly egressed tachyzoites of the type II (Prugniaud) cystogenic strain (data not shown) and immunoprecipitations targeting the YRS and MRS subunits of the MARS complex subsequently confirmed the composition of the complex (Figure 3). It can also be seen from Figure 3 that the MRS and YRS components were the least abundant (when not the target of immunoprecipitation) and that the level of Tg-p43 and ERS was always higher than that of QRS in the isolations. Degradation products were also detected in eluates of the least abundant subunit, MRS, the only case where this was detected. The detection of any of the component subunits was not affected by salt concentrations, up to 500 mM of either potassium chloride or ammonium acetate, during the washings stages of the affinity purifications.

**Figure 4:**
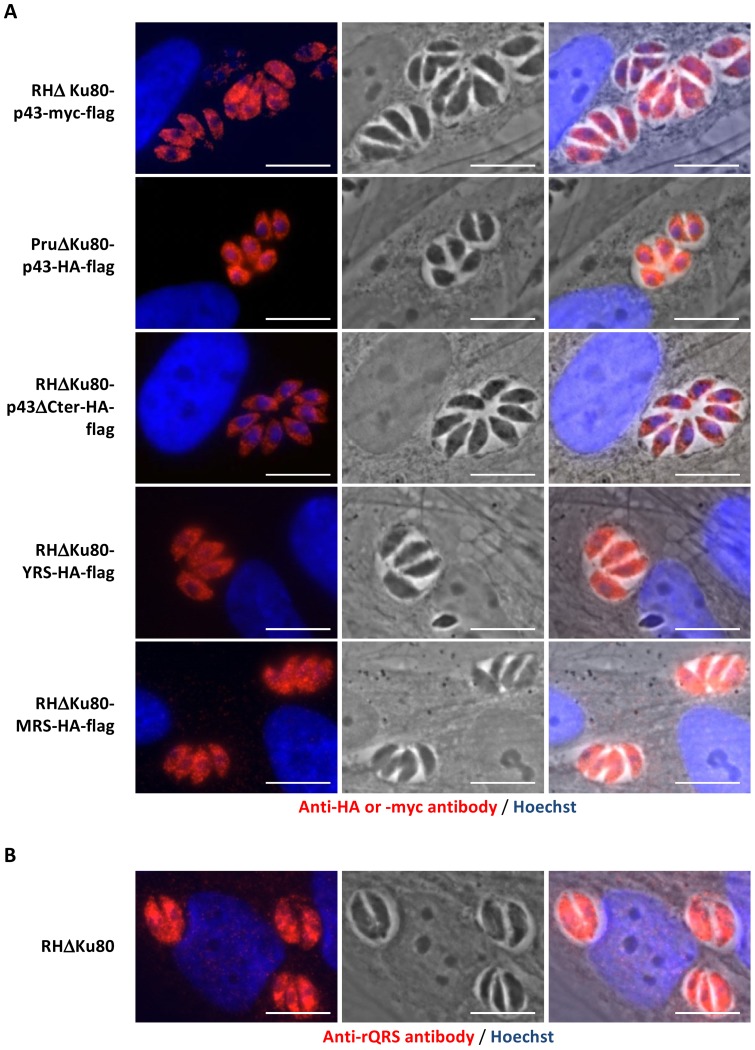
Localization of the Tg-MARS complex. (A) Fluorescent, light, and overlay of the two channels of endogenously tagged subunits of Tg-MARS visualised by *in situ* immunofluorescence in intracellular parasites with anti-Myc or anti-HA (red) antibodies and Hoechst DNA-specific dye (blue). (B) Fluorescent, light, and overlay of two channels of the QRS subunit of Tg-MARS visualised by *in situ* immunofluorescence in intracellular parasites with an anti-rQRS (red) antibody and Hoechst DNA-specific dye (blue). Scale bar  = 10 µm.

Unfortunately, it was not possible to generate viable *Toxoplasma* parasite strains expressing C-terminal tagged version of the ERS or QRS proteins. Instead we raised antibodies against heterologously-expressed QRS and immunofluorescence experiments using these identified an exclusively cytoplasmic location for QRS in agreement with the localizations of Tg-p43-HA-FLAG, MRS-HA-FLAG, and YRS-HA-FLAG (Figure 4A and Figure 4B).

### The N-terminal GST C-terminal like domain of Tg-p43 is sufficient to mediate complete assembly of the MARS complex

**Figure 5:**
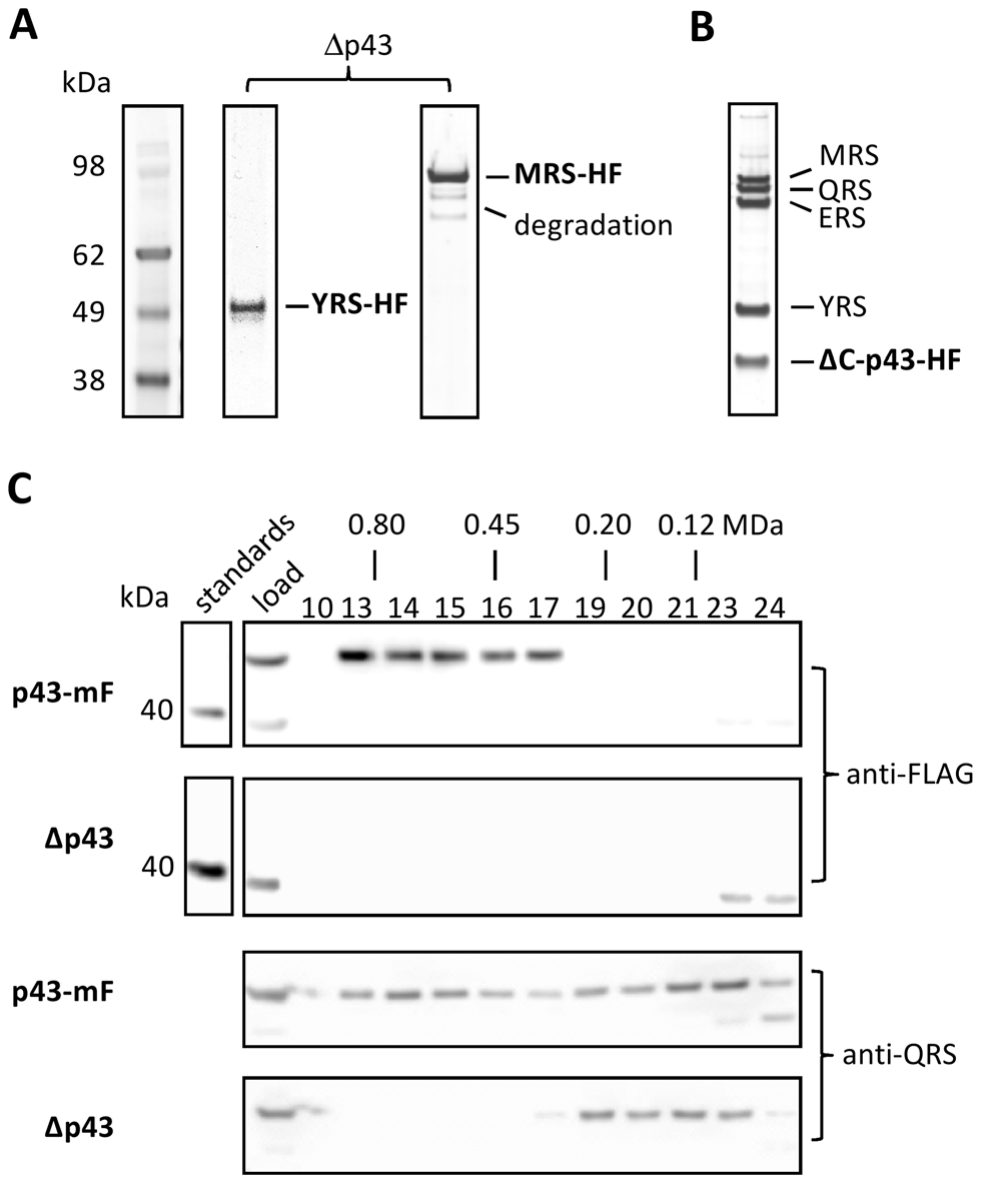
Dependence of MARS complex assembly on the scaffold protein Tg-p43. (A) Silver-stained PAGE gel of immunoprecipitations of HA-FLAG-tagged (HF) YRS and MRS subunits in Tg-p43 knockout strains. (B) Isolation of the MARS complex by immunoprecipitation of a C-terminal-truncated (ΔC) form of the Tg-p43 protein (a.a. 1–296). (C) Western blots of eluted fractions from SEC analyses of crude cell lysates of Tg-p43-containing and knock-out strains (all RHΔ*ku80*). Corresponding fractions (numbered above the first gel image) are vertically aligned and their solution molecular weights, as determined by calibration with high molecular weight standards, are also given. Molecular weight standards are shown for the anti-FLAG Western only as a reference for evaluation of the knock-out blot.

Tagging and immunoprecipitation experiments of select subunits were then carried out in parasite strains lacking *Tg-p43* (Figure S1) to confirm the predicted role of Tg-p43 in complex assembly and to identify any sub-complexes formed in its absence. The deletion of *Tg-p43* resulted in total disruption of the complex as evidenced by the absence of any binding partners in the eluates of both YRS and MRS HA-FLAG-tagged subunits (Figure 5A) from *Tg-p43* knockout strains (Δp43). Additional bands were visible in the latter case but MS-MS analyses confirmed that these were the degradation products seen previously rather than a sub-complex of MRS with other aaRS of *Toxoplasma* MARS.

In addition to the knock-out of Tg-p43, a tagged mutant lacking the C-terminal half of the protein i.e., only containing residues 1 to 296, was created to ascertain whether both domains of the protein are responsible for complex assembly. Immunoprecipitations of the Tg-p43ΔC-HA-FLAG protein showed the same pattern of co-eluting partners (as identified by MS-MS) thus confirming that the N-terminal GST-like domain alone is sufficient for assembly of the *Toxoplasma* MARS component subunits (Figure 5B).

Because it was not possible to create a tagged QRS protein, size-exclusion chromatography of cell-free preparations of Tg-p43-containing and *Tg-p43* KO strains was used to determine whether the recruitment of QRS to the MARS complex is mediated by Tg-p43 (Figure 5C). Western blot analysis using anti-QRS polyclonal antibodies clearly identified a high-molecular weight fraction, corresponding to a calibrated molecular weight between 1.0 and 0.3 MDa, in p43-containing tachyzoites but not in the KO strain.

### *Toxoplasma* MARS complex displays significant heterogeneity in solution

**Figure 6:**
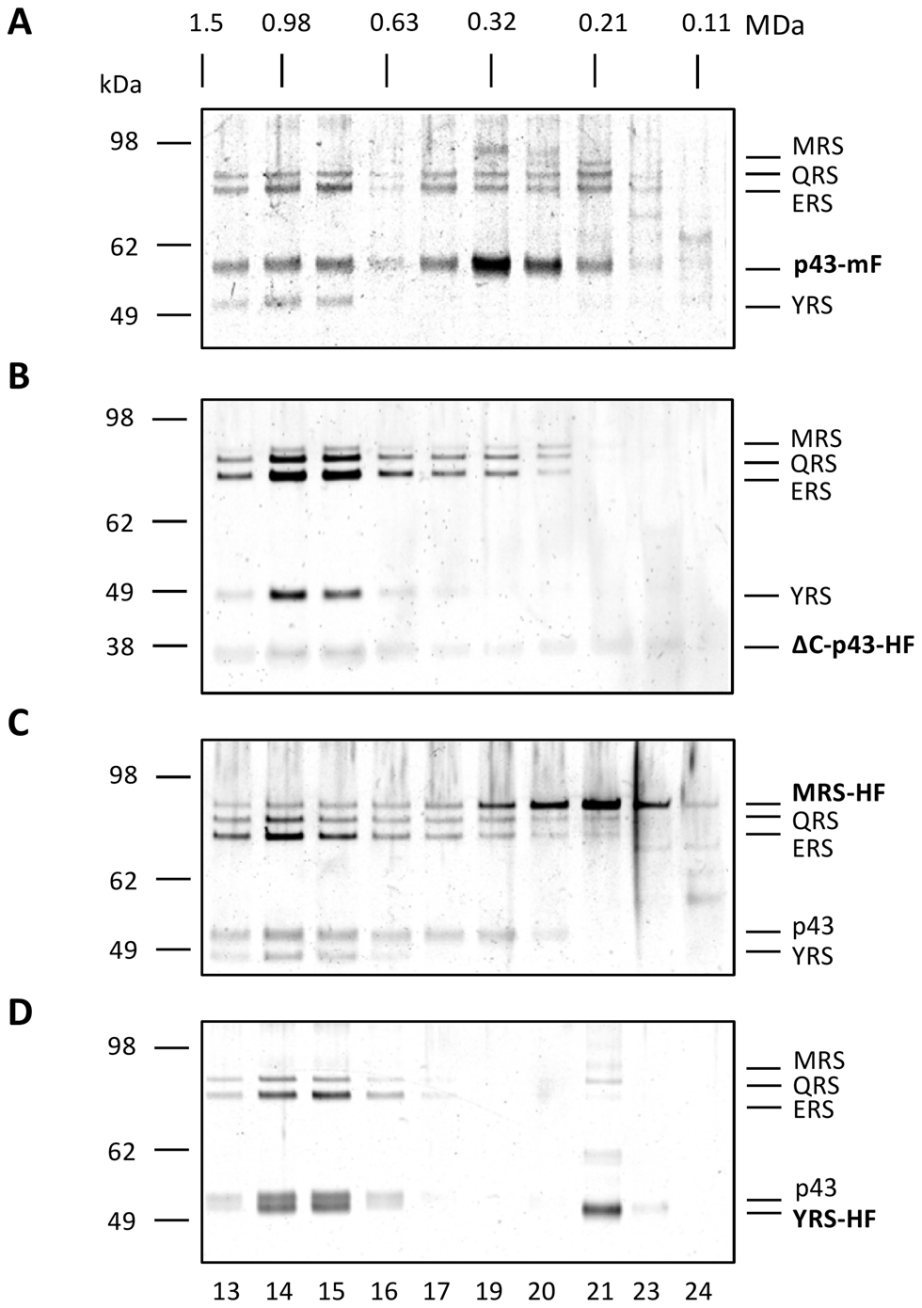
Compositional and size heterogeneity of MARS complex populations. Silver-stained PAGE gel of eluted fractions of immunoprecipitated MARS complexes separated by SEC. Each panel represents a separate SEC analysis of MARS complexes purified from strains harbouring C-terminal tags on different subunits of the complex: (A) Myc-FLAG-tagged Tg-p43 (mF = Myc-FLAG), (B) HA-FLAG-tagged C-terminal-truncated (ΔC) form of the Tg-p43 (residues 1–296) (HF = HA-FLAG), (C) HA-FLAG-tagged methionyl-tRNA synthetase, and (D) HA-FLAG-tagged tyrosyl-tRNA synthetase. Corresponding fractions (numbered below each gel image, and calibrated by the elution peak of the FLAG peptide) are vertically aligned and their solution molecular weights, as determined by calibration with high molecular weight standards, are given. Grey-levels of the images were adjusted to enhance the contrast but no bands were masked by this process.

The large upper size limit of the *Toxoplasma* MARS complex was confirmed by extensive size-exclusion chromatographic separations of FLAG-tagged material which also revealed a distribution of molecular weight sub-species (Figure 6). The size distribution of proteins co-eluting with the scaffold protein, Tg-p43-Myc-FLAG, displayed an almost bimodal distribution of species with a high molecular weight peak centred on 1.0 MDa and a low molecular weight peak occurring around 0.3 MDa, with lower concentrations of subunits found in the intervening fractions (Figure 6A). The lower molecular weight peak, the position of which (250 kDa) was similar to the measurements from recombinant Tg-p43 (Figure 2B), contained the largest fraction of Tg-p43. Most interestingly, however, all of the detectable YRS protein was found in the high molecular weight fractions (Figure 6A) and similar results were obtained from separations of Tg-p43ΔC-HA-FLAG (Figure 6B) and MRS-HA-FLAG (Figure 6C). This preferential association with the high molecular weight complexes was subsequently confirmed by isolations of YRS-HA-FLAG-tagged material (Figure 6D) which were highly enriched in this species with a conspicuous absence of intermediate and low molecular weight species (except for some presumably dimeric YRS). The Tg-p43ΔC-HA-FLAG complex displayed a similar enrichment but with a broader distribution of QRS and ERS proteins occurring towards lower MWs (Figure 6B).

### Flexible domains surround a central ring-like core in the *Toxoplasma* MARS complex

**Figure 7:**
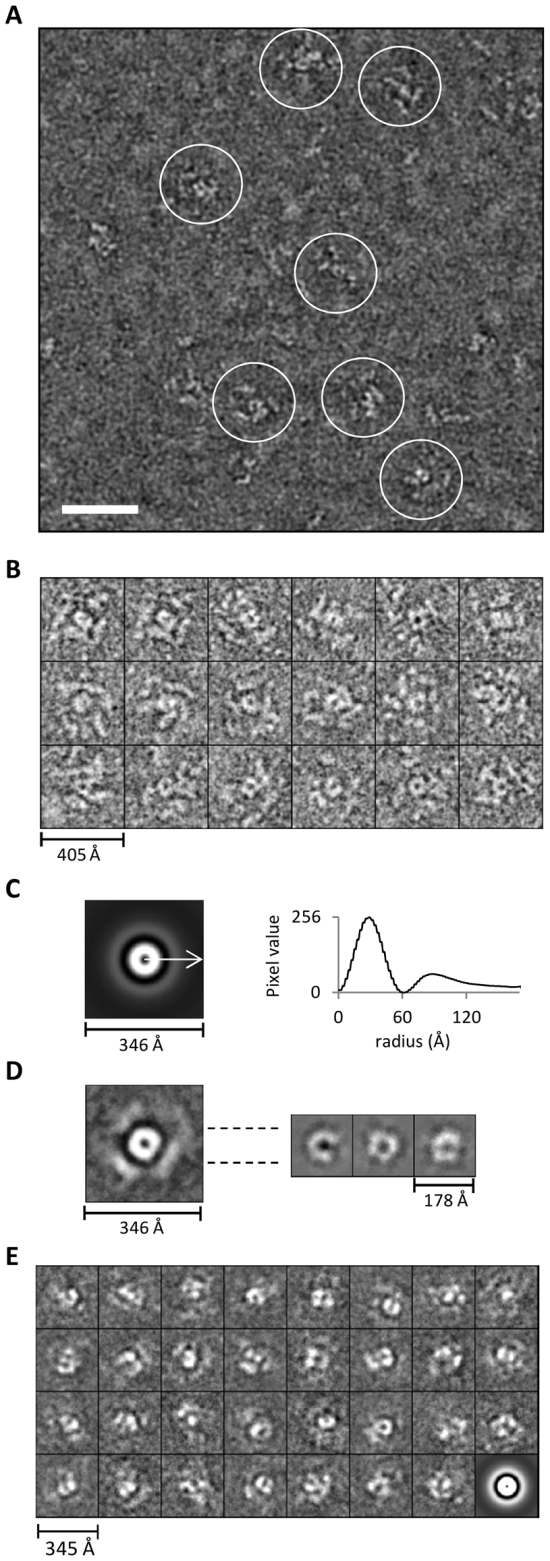
Appearance of the MARS complex as revealed by electron microscopy. (A) Electron micrograph of negatively-stained YRS-HA-FLAG MARS complexes. The scale bar represents 50 nm. (B) Subset of representative propeller views (18/137) of negatively-stained immunoprecipitated YRS-HA-FLAG MARS complexes windowed from images such as shown in (A). (C) Left – Rotational average of all views (1030 particle including the 137 propeller views) following pre-centring. Right – Corresponding radial profile from which the central core (120 Å) and peripheral domain's (240 Å) maximum average diameters can be determined. (D) An average image of aligned representative particles (137 propeller views from B) based on the entire image is shown on the left. On the right, three different class averages (homogenous sub-classes) generated by re-aligning only the central regions of the 137 views on the left are presented. (E) Reference-free class averages (homogenous sub-classes) derived from classification of all windowed particles (1030 in total) not just the propeller views shown in (B). The last image is the rotational average of all particles that went into classification.

The poor homogeneity of the Tg-p43-Myc-FLAG- and MRS-HA-FLAG-tagged MARS complexes was confirmed by electron microscopy investigations which failed to identify a species of particle representative of the large molecular weight MARS complex. Unfortunately, attempts at imaging the various molecular weight species separated during SEC were no better due to the apparent susceptibility of the MARS complex to the large dilution effects imposed during separation (data not shown). In order to circumvent this heterogeneity, subsequent EM studies focused on the concentrated preparations of immunoprecipitated YRS-HA-FLAG-tagged material. Large and well separated particles were clearly visible in negatively-stained micrographs of these samples (Figure 7A) but these particles were not very distinct due to the extended nature of their substructures. These domains, although clearly separated from each other, were not arranged as the dense globular structures expected for large macromolecular complexes.

Careful analysis of the images led to the identification of a subset of particles displaying a consistent view of the large particles, or a species thereof (Figure 7A and 7B). In these views it is clear that particles have the overall appearance of a propeller-like structure with peripheral sub-structures radiating from a central ring-like core. Averaging of centred but not rotational aligned images clearly shows that the central ring, with a maximum diameter of 120 Å, is the most conserved feature between particles (Figure 7C). The arrangement of the peripheral substructures of the particles, however, differ not only in their distance from the core, with a maximum diameter of 240 Å, but also in their arrangement around the circumference leading a much weaker ring of averaged density (Figure 7C). This is further exemplified by the rotational alignment and averaging (compared to only centring) of images which resulted in the appearance of weak and diffuse density arranged in spatially separated regions around the periphery of the particle (Figure 7D – left). The core region, on the other hand, produces more homogenous class averages (sub-classes of aligned views), which display subdivisions consistent with an oligomeric arrangement of proteins, when only this central region is aligned and averaged (Figure 7D – right). Similar alignments and classifications of all the particles in the micrographs (not just the propeller-like views) confirmed this structural flexibility by showing a strong dominance of the central region in the highly heterogeneous averages (Figure 7E).

## Discussion

### Presence and features of Tg-p43

Our preliminary isolations of tagged endogenous Tg-p43 confirmed the bioinformatics analyses of the *Toxoplasma* genome [35] which predicted the presence of a protein with both an N-terminal, all-alpha helical, glutathione-S-transferase C-terminal-like domain and a C-terminal EMAPII–like tRNA binding domains. Our results however showed that the latter domain is not cleaved to become an extracellular cytokine, as in the case of the human p43 protein [36], [37]. Furthermore, the human p43 protein does not possess a GST C-terminal like domain. Instead, these motifs are contributed by the p38 and p18 accessory proteins. In this sense, the domain structure of Tg-p43 more closely resembles the yeast Arc1p protein which is also a hybrid scaffold protein containing sequence features resembling different regions of the human AIMPs. This similarity is reflected at the sequence level where these proteins share 20 and 25% identity between their matching GST-C and tRNA-binding domains, respectively. In comparison, the divergence is much larger between the Tg-43 GST domain and the human p43 and p38 proteins which only share 17% sequence identity.

**Figure 8:**
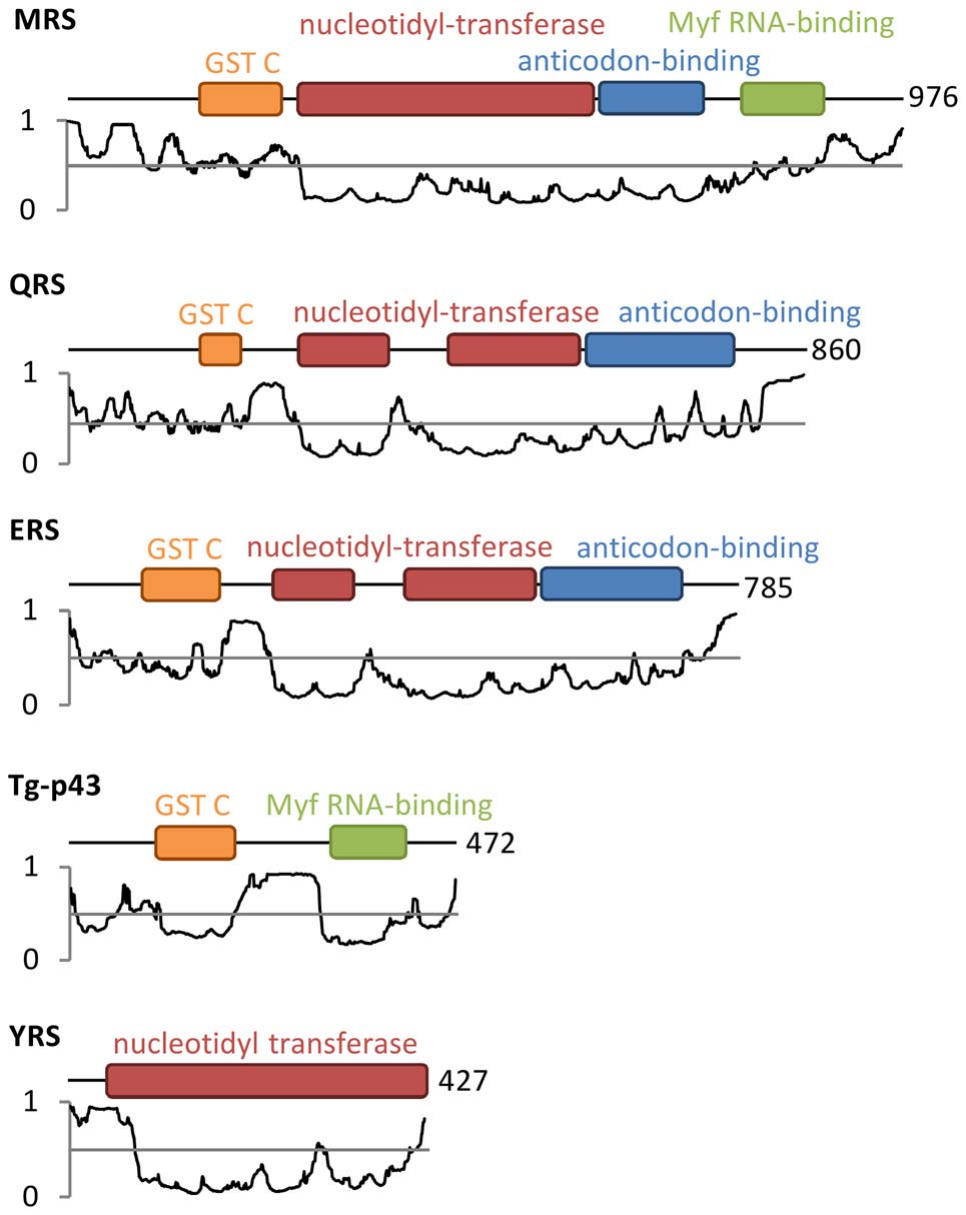
Disorder potential and domain structures of *Toxoplasma* MARS complex subunit proteins. Genesilico MetaDisorder2 disorder predictions [28] for each peptide are displayed below domain arrangement schematics derived from NCBI conserved domain searches [24]. Values above 0.5 are predicted to be disordered (coloured red) and values below 0.5 correspond to folded domains (coloured green). The number of residues in each protein is given after each schematic.

The remainder of the Tg-p43 sequence shares very little homology with known proteins and was predicted to be largely disordered based on the percentage of highly charged and polar residues (Figure 8). The results of our circular dichroism measurements confirmed that 38% of the protein was unfolded thereby partially explaining the SEC results which suggested a far larger hydrodynamic radius than expected from molecular weight considerations. The fact that Tg-p43 was retained by filters with MWCO of 100 kDa and its susceptibility to rapid cross-linking as a dimeric species however also suggests that a tendency to oligomerise may be contributing to the unexpected solution behavior of Tg-p43.

### Composition and location of the MARS complex

By demonstrating that a subset of aaRSs (MRS, QRS, ERS, and YRS) are binding partners of the Tg-p43 protein, our affinity purification experiments confirmed the predicted role of the protein in MARS complex formation and our immunofluorescence data confirmed in parallel that these components occur exclusively in the cytoplasm. Inspection of the sequences of these subunits shows that they possess large N-terminal extensions that are predicted to be partially disordered and in all cases, except for the YRS component, these regions are predicted to encode a GST-C terminal like domain (Figure 8). We believe that this group of five distinct proteins represent the stable cytoplasmic *Toxoplasma* MARS complex because immunoprecipitations targeting the MRS and YRS subunits gave identical results to the Tg-p43 isolations (Figure 3). The presence of a single scaffold protein and the small number of enzymes in the *Toxoplasma* MARS complex, together with the fact that they are all class I aaRS enzymes [38], suggests further similarities with the yeast MARS complex wherein only ERS and MRS are present with Arc1p. In contrast, human and other metazoan complexes contain three scaffold proteins and comprise representative members of aaRSs from both classes. This, as far as we know, is also the first report of a YRS enzyme being found in a MARS complex.

One theory for the role of the MARS complex, put forward to explain the presence of ERS and not QRS in the yeast complex, is to recruit additional tRNA-binding domains to aaRSs that lack them [15]. The presence of MRS, which also contains a myf-tRNA binding domain, in the *Toxoplasma* MARS complex is therefore puzzling if this is also the driver for inclusion in the *Toxoplasma* complex. The choice of aaRSs and the exact function of the MARS complex in *Toxoplasma* therefore remains a mystery especially in light of our genetic analyses in both virulent (type I) and non-virulent but cystogenic, (type II) *T. gondii* strains showing that Tg-p43 is not essential for viability or pathogenesis of the parasite. Together with what is known about the non-essential nature of AIMPs in yeast [12]] and humans [9], our results further suggest that the contribution of the MARS complex to post-transcriptional gene control must be subtle.

### Assembly of the complex

Based on the similarity of the overall domain structures and the degree of homology between the Tg-p43 and the yeast Arc1p proteins it was assumed that assembly of the *Toxoplasma* complex would be driven by similar mechanisms. The associations between the yeast Arc1p protein and its ERS and MRS partners have been attributed to pairwise dimerizations occurring at distinct interfaces in the GST domain of the scaffold protein [21]. The presence of MRS, QRS, and ERS, all of which possess GST-like motifs, in the *Toxoplasma* complex was therefore not surprising but the inclusion of YRS was unexpected as its N-terminal extension is predicted to be highly disordered. Until more structural information is available for the N-terminal region of the *Toxoplasma* YRS protein, it remains to be seen in the GST motif is structurally conserved despite this apparent sequence divergence.

We were able to reveal a direct association of MRS and YRS with the Tg-p43 and we subsequently showed that only the N-terminal region of this scaffold protein, harbouring the GST-motif, was necessary for the association. Although it was not possible to demonstrate direct interactions for the QRS and ERS proteins, because modifications to these genes compromised the viability of the parasite, we did show that the presence of QRS in the high-molecular weight fraction depended on Tg-p43. We cannot however exclude the possibility that QRS is recruited to the MARS complex through an interaction with ERS. Dissecting the interactions of these essential proteins will require biophysical characterisation of pairwise interactions in native MARS complex subassemblies or recombinant proteins.

### Heterogeneity in MARS composition and sizes

The results of our SEC studies showed that the *Toxoplasma* MARS complex exhibited various degrees of size and compositional heterogeneity depending on which subunit was the target for affinity purification. Both the Tg-p43-Myc-FLAG and MRS-HA-FLAG preparations displayed the broadest distribution of molecular weight species, between 0.3 and 1 MDa, which also differed significantly in the relative abundance of their component subunits. By contrast, preparations from YRS-HA-FLAG and ΔC-terminal-Tg-p43-HA-FLAG strains were highly enriched in the high molecular weight fractions leading us to believe this 1MDa species corresponds to the intact *Toxoplasma* MARS complex. Even if the fact that both YRS and MRS are expected to be dimeric [19], [39] is considered along with the possibility that Tg-p43 could be oligomeric, the 1 MDa size estimate is far larger than expected. Such considerations predict a minimum molecular weight of 591 KDa for a complex composed of two copies of Tg-p43, MRS, and YRS, and a single copy of ERS and QRS. In any regard, if this species represents the fully-assembled complex, then the lower molecular weight distributions and the differing levels of heterogeneity must be interpreted as resulting from differences in the affinity of the subunits for the labile complex. The exclusive presence of YRS and it relatively low abundance in the high molecular weight complex could therefore be understood to reflect its weak association with Tg-p43. Such affinity/stability considerations make absolute size estimations of the various sub-complexes difficult and this is especially true considering that the sequences of the component subunits are predicted to contain some degree of disorder which can be expected to manifest as more extended and flexible domain organisations.

### Ultrastructure of the MARS complex

Together with the results of our EM analyses which identified both compositional and structural heterogeneity, these hydrodynamic results suggest that previous concerns about MARS complex stability and sensitivity to preparation conditions [40] are justified in this instance too. By targeting the YRS subunit for affinity purification of *Toxoplasma* MARS complexes however we were able to overcome these issues thereby allowing us to directly visualize these structures in the microscope. Although it was possible to recognize consistent features in a fraction of the particles, the most telling result was in fact the absence of a large distinct consensus particle view, as expected for a 1 MDa globular assembly. Instead, image analysis revealed a “propeller-like” structure wherein a central ring-like core, exhibiting clear sub-domains, is surrounded by highly flexibly and spatially separated substructures. Not only does the extended arrangement of these subunits explain some of the heterogeneity exhibited by the MARS complex as arising from their intrinsic flexibility but it also argues in favour of a smaller absolute molecular weight estimate for the complex which we believe exhibits a larger than expected hydrodynamic radius due to the intrinsic disorder present in the component subunits. Such behaviour has now been conclusively proven for the human MARS complex and is thought to be necessary for allowing access of bulky tRNA substrates to the active sites of independent component enzymes [41]. This flexibility has important implications for future structural studies which we believe will need to focus on the central core of the *Toxoplasma* MARS complex in order to provide a detailed understanding molecular interactions underpinning the assembly of this complex in both apicomplexa and higher eukaryotes.

## Supporting Information

Figure S1 **Generation and confirmation of p43KO in RHΔKu80 and PruΔKu80 strains.** (A) Schematic of the Tg-p43 locus (not drawn to scale). Double homologous recombination between the knockout construct and genomic DNA replaces *p43* with the muted *DHFR* gene, which was used for positive selection. Primers used to confirm a knockout are shown (F1, OL1F, OL1R, R1, F9, R9). (B) After transfection of *T. gondii* in RHΔKu80 or PruΔKu80 strains with the p43KO fragment, parasites were cloned by limiting dilution, and genomic DNA was isolated. This genomic DNA was then used as template in a PCR reaction to amplify the *p43* locus (F1 and R1 amplify a 6.9 kb fragment in knockout strains and a 9.3 kb fragment in non-mutant strains; F9 and R9 amplify a 1.1 kb fragment only in non-mutant strains). Two bands that are only present in a successful knockout were also amplified (F1 and OL1R amplifies a 2.1 kb fragment; OL1F and R1 amplifies a 2.0 kb fragment).
(TIF)

Figure S2 **Deletion of Tgp43 does not alter the lethality of type I or II strains in mice.** Virulence of type I RHΔku80Δp43 (A) and type II PruΔku80Δp43 (B) strains were compared to the parental strains RHΔku80 and PruΔku80, respectively, in Swiss mice. Mice were inoculated with 10^2^ or 10^6^ (lethal inoculum) tachyzoites of type I and type II strains, respectively, by intraperitoneal injection, and survival was monitored. The number of infected animals is indicated in the legend.
(TIF)

Table S1 Primers and vectors used for genetic manipulation of *T. gondii* and for heterologous recombinant expression of *T. gondii* proteins.
(PDF)
