# Supplementary figures and images for: Assembly of the Novel Five-Component Apicomplexan Multi-Aminoacyl-tRNA Synthetase Complex Is Driven by the Hybrid Scaffold Protein Tg-p43
Source: PLoS One. 2014 Feb 20;9(2):e89487. doi: 10.1371/journal.pone.0089487 (PMC3930741; doi:10.1371/journal.pone.0089487)

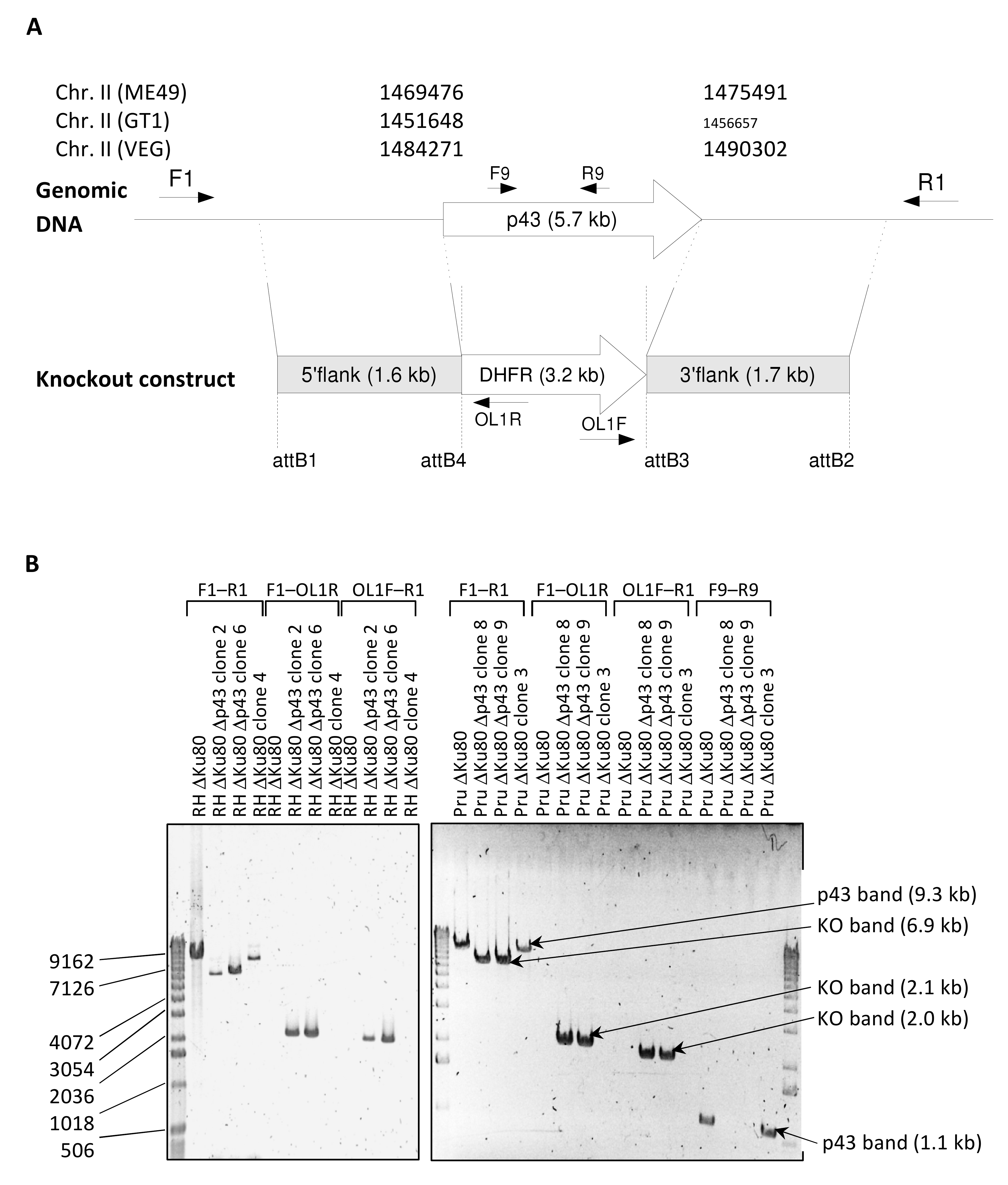

Supplement: Figure S1 — Generation and confirmation of p43KO in RHΔKu80 and PruΔKu80 strains. (A) Schematic of the Tg-p43 locus (not drawn to scale). Double homologous recombination between the knockout construct and genomic DNA replaces p43 with the muted DHFR gene, which was used for positive selection. Primers used to confirm a knockout are shown (F1, OL1F, OL1R, R1, F9, R9). (B) After transfection of T. gondii in RHΔKu80 or PruΔKu80 strains with the p43KO fragment, parasites were cloned by limiting dilution, and genomic DNA was isolated. This genomic DNA was then used as template in a PCR reaction to amplify the p43 locus (F1 and R1 amplify a 6.9 kb fragment in knockout strains and a 9.3 kb fragment in non-mutant strains; F9 and R9 amplify a 1.1 kb fragment only in non-mutant strains). Two bands that are only present in a successful knockout were also amplified (F1 and OL1R amplifies a 2.1 kb fragment; OL1F and R1 amplifies a 2.0 kb fragment). (TIF) [file pone.0089487.s001.tif]

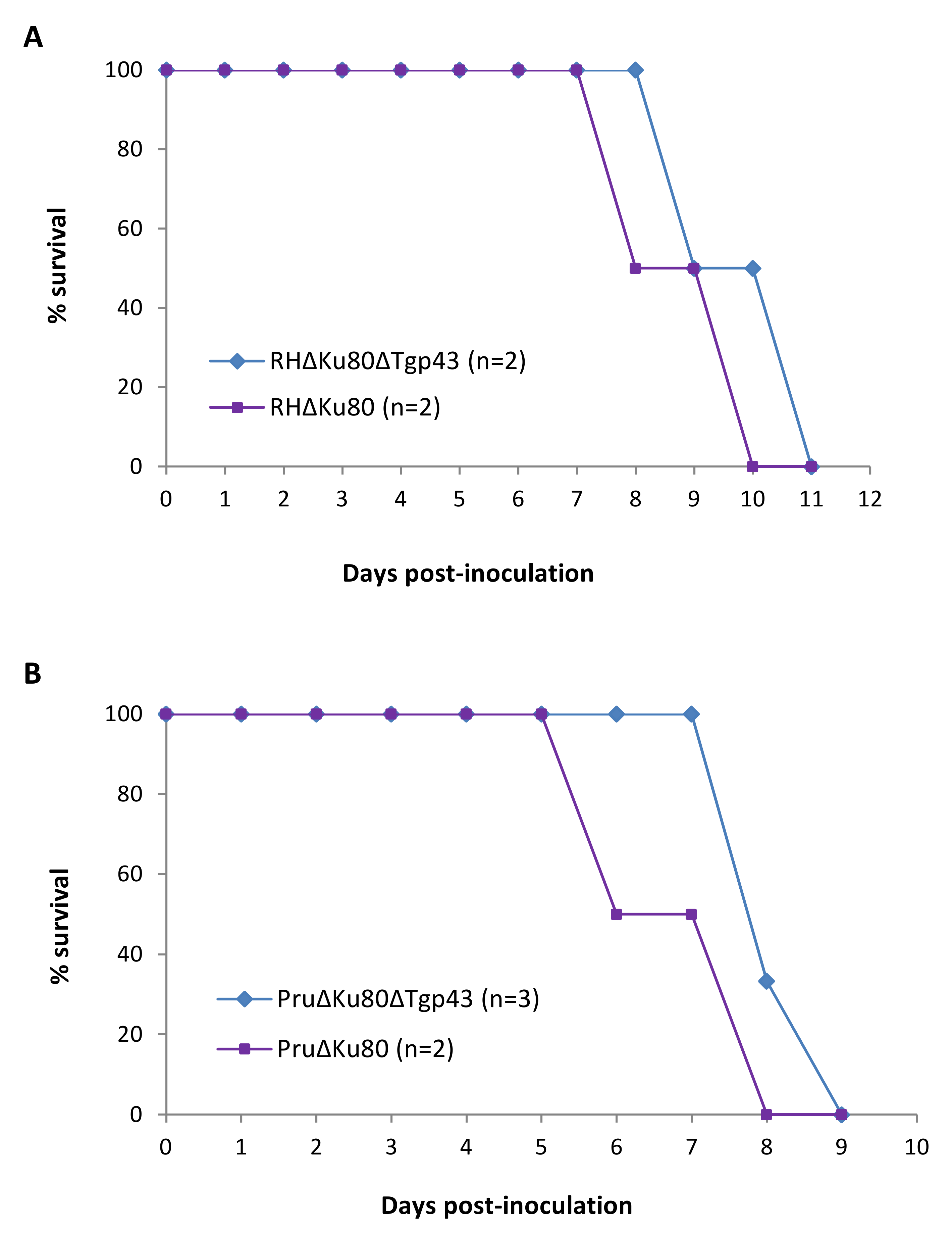

Supplement: Figure S2 — Deletion of Tgp43 does not alter the lethality of type I or II strains in mice. Virulence of type I RHΔku80Δp43 (A) and type II PruΔku80Δp43 (B) strains were compared to the parental strains RHΔku80 and PruΔku80, respectively, in Swiss mice. Mice were inoculated with 102 or 106 (lethal inoculum) tachyzoites of type I and type II strains, respectively, by intraperitoneal injection, and survival was monitored. The number of infected animals is indicated in the legend. (TIF) [file pone.0089487.s002.tif]
