# Supplementary material for: Assembly of the Novel Five-Component Apicomplexan Multi-Aminoacyl-tRNA Synthetase Complex Is Driven by the Hybrid Scaffold Protein Tg-p43
Source: PLoS One. 2014 Feb 20;9(2):e89487. doi: 10.1371/journal.pone.0089487 (PMC3930741; doi:10.1371/journal.pone.0089487)
Supplement: Table S1 — Primers and vectors used for genetic manipulation of T. gondii and for heterologous recombinant expression of T. gondii proteins. (PDF) [file pone.0089487.s003.pdf]

Supporting Table 1: Primers and vectors used for genetic manipulation of *T. gondii* and for heterologous recombinant expression of *T. gondii* proteins.

Vector	Amplified region	Primer name	Primer sequence	Amplified fragment size
pLIC vectors - expressed in *T. gondii*	*Tgp43*	LIC066670-F	tacttccaatccaatttagcccgacgattcgcgccaggacaccctgtg	2299 bp
		LIC066670-R	tcctccacttccaattttagcagagatggtgcctccgacgatagagtcac	
		LICp43delta-F	tacttccaatccaatttagctcctttttagttaaaacgacgacgctttc	1687 bp
		LICp43delta-R	tcctccacttccaattttagctccacctttcgacgaaccgggtgtgctctg	
	TgYRS	LIC103260-F	tacttccaatccaatttagccgcaggcatggacatgcaaaacgtccgc	2121 bp
		LIC103260-R	tcctccacttccaattttagctttcgtcactttgtatgctttaatttgctg	
	TgMRS	LIC289300-F	tacttccaatccaatttagcgacttgcgattcgacgaagacgccttgac	1157 bp
		LIC289300-R	tcctccacttccaattttagcaagcgcgagagaaaggcgcccttggcag	
	Upstream flanking region of *Tgp43*	attB1-066670	ggggacaagtttgtacaaaaaagcaggctctgaagcagctcagccgcagggttgcac	1609 bp
		attB4-066670	ggggacaactttgtatagaaaagttgggtggcgcaagacaaagatgcatgcttgcg	
	Downstream flanking region of *Tgp43*	attB2-066670	ggggaccactttgtacaagaaagctgggtacctcgatgttcacaatgataatgtgg	1719 bp
		attB3-066670	ggggacaactttgtataataaagttgcaatggactaacgtgttgcttccagc	
pETDuet-1 - expressed in *E.coli*	*Tgp43* (*TgVEG_053640* residues 208-679)	TgP43-Fwd-NcoI	catgccatggaactgatttatgatagcacaccgg	n/a
		TgP43-Rev- His-BamHI	cgcggatccttaatgatgatgatgatgatgagcaccagcaccgctgatggtgccaccaacaatgctatcgcaaaaaac	n/a
pcDNA4 - expressed in HEK293	*Tgp43* (*TgVEG_053640* residues 208-679)	p43-pcDNA4-Cter-Fwd	tggctggtgctggtgcccatgaactgatttatgatagcacaccggcagc	n/a
		p43-pcDNA4-Cter-Rev	gctccggctcctgccccagcgctgatggtgccaccaacaatgctatcgc	n/a
		p43-pcDNA4-Rev	gctccggctcctgccccagcttagctgatggtgccaccaacaatgctatcgc	n/a
